# Restoration: Implant with Devastated Platform through Metal Post

**DOI:** 10.1155/2017/3150656

**Published:** 2017-02-13

**Authors:** Luna Salinas Tatiana, Del Valle Lovato Juan

**Affiliations:** Central University of Ecuador Dental School, Quito, Ecuador

## Abstract

*Case Presentation. *Implant prostheses are a successful treatment for replacing missing teeth. However, this treatment modality can have biological and mechanical complications causing serious problems for the dentist, as demonstrated in this clinical case. The patient presented with a fractured screw and a severely damaged implant hex connection that corresponded to the second premolar, upper left, stating that she unsuccessfully tried to remove the prosthetic screw, which was most likely to have been loose. After clinical and radiographic review, it was decided to remove small fragments of the fractured prosthetic screw inside the implant head. Removal by conventional methods was unsuccessful but was eventually achieved through use of a bur. Then it was possible to make a cast post (gold-palladium) and develop a fixed prosthesis (silver-palladium), which were attached with luting cement. A cast post (gold-palladium) was made and a fixed prosthesis was developed (silver-palladium), which were attached with luting cement, the same ones that can present mechanical complications such as fractures between the third and fourth thread of the implant, loosening of the abutment, and/or the prosthetic screw in individual crowns, most frequently in partially edentulous patients, mainly in the premolar and molar regions of the maxilla.* Conclusion*. Therefore the present technique used in this case is very simple, noninvasive, and useful to readers.

## 1. Introduction

Since Branemark introduced the concept of osseointegration, dental implants have been successfully used as a viable treatment for fully and partially edentulous patients [1]. According to Zarb and Schmitt [2] and Wismeyer et al. [3] implant fracture is a rare but significant complication, most of the fractures occurred between the third and fourth thread of the implants. Several authors [4–8] concluded that the loosening of the abutment screw ranged from 2% to 45% and that there was a difference in the incidence of loosening between types of prosthesis; the highest rate was found with single crowns following and overdentures.

The most common complication for a single crown was a prosthetic screw and abutment screw loosening [9], but the prosthetic complications will depend on the number of implants available, size, and arrangement and may cause long-term marginal bone loss, fractures metal fatigue, and/or loss of osseointegration [10, 11].

Hurson [12] states that the nature of loosening is complex because a variety of patterns and occlusal masticatory forces, The clinical studies indicate that between 5% and 45% of loosening or fracture of the components of the implant prosthesis occurs in the first year, Gupta et al. [13].

According to Nergiz et al. [14], screw is the smallest and weakest part between the implant components; therefore it may be lost or broken before other components. Besides the implant systems have such antirotation component as an internal or external hexagon so implants that are not protected against rotation present higher percentage of complications [14].

Jemt [15] states that the screws are typically designed to be the weakest link in the implant-prosthetic system, loosening being an early sign of overload. According to W. Becker and B. E. Becker [16] this loosening in fixed prostheses connected with external hex implants is a phenomenon that occurs most frequently in partially edentulous patients.

Parafunctional habits can be a risk factor related to implant fracture and screw loosening and can create uncontrolled and excessive occlusal loading forces [17]. Indeed some authors state that both the centric and eccentric bruxism can lead to overloading of the implant and metal fatigue as a result of physiological changes of the patient [18, 19].

A higher frequency of screw loosening has been reported for replacement of single crowns in the premolar and molar area than in the anterior region and three times more in the maxilla compared with the mandible [20]. The posterior maxilla had a success rate of 91.4% compared with the maxilla (97%), the posterior mandible (96.3%), and the anterior mandible (97.9%) [21].

Once fracture has been diagnosed, it is possible to proceed to extraction, beginning with the simplest and most conservative method, and trying to respect, as far as possible, the implant head, external hex, and internal thread. Any change in these areas can lead to limitations in future prosthetic use. The methods used to recover the broken fragments or screw are determined according to the location above or below the head of the implant. If a cap screw fracture is above the head of the implant, an explorer, a straight, or a hemostatic probe can succeed, and the tip of the instrument is carefully moved in the opposite direction clockwise on the surface of the segment screw according to Satwalekar et al. [22]. If that procedure is unsuccessful in removing the fragment, Eckert et al. [23] have proposed applying a round bur at high speed to the head of the broken screw and another method is to make a notch in the head fragment, if possible, to attempt to remove the implant fragment by using a screwdriver in reverse. In case of implant fracture, there are two options: (1) complete removal of the implant fractured using explantation drills and (2) the use of the fractured implant in order to place a new prosthesis [24]. Some implant manufacturers offer a kit for this purpose, including a rotary tool to smooth the edges of the fracture and an instrument to create a new internal thread for the implant. Work of Goiato et al. [25] proposes a third option, which is to leave the submerged implant. If the implant is again rehabilitated, noble metal alloys, including gold, palladium, silver, and titanium, should be used. Proper selection and handling of these alloys are essential because the prosthetic restoration and longevity go hand in hand with implants [26, 27].

Finally, although the frequency of fractures of implants is low, treatment planning should include avoiding occlusal overload, in some cases using an occlusal splint to protect the restorations [28].

This report describes the rehabilitation of an implant with screw fracture and severely damaged hex by a cast post.

## 2. Case Presentation

**Figure 1:**
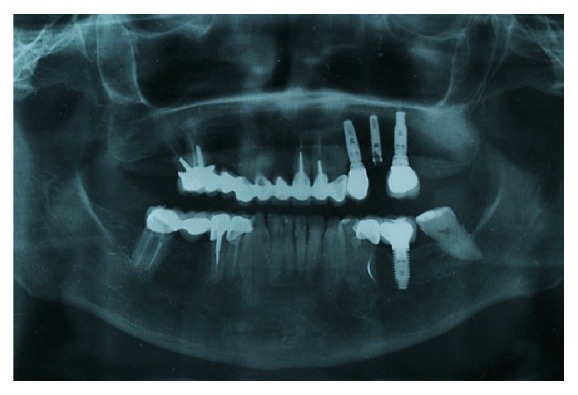
Panoramic radiograph. Implant (2.5) with fracture screw and hex devastated platform.

A 66-year-old female patient who takes bisphosphonates for osteopenia presented at the dental practice in the Postgraduate School of Oral Rehabilitation, Central University of Ecuador, with fracture of the implant prosthetic screw and damaged hex platform (Figure 1).

**Figure 2:**
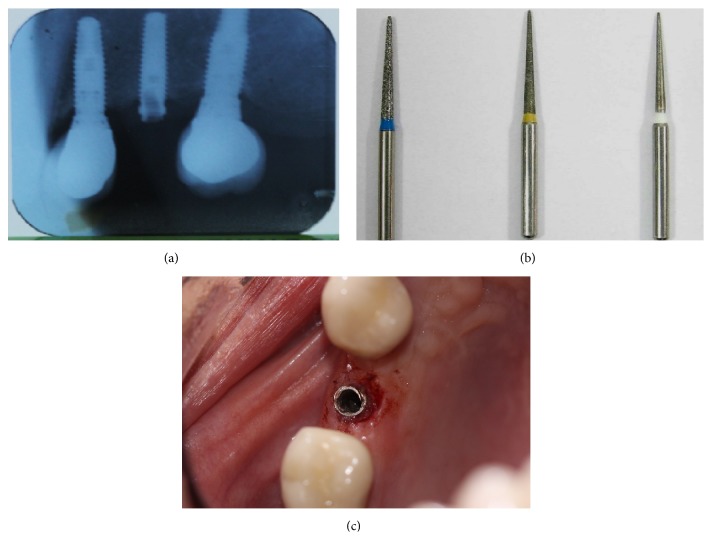
Removal of fracture fragment: (a) radiograph; (b) fissure bur was used to remove the fractured screw; (c) occlusal view.

The first step was to attempt extraction of the fragment, beginning with the most conservative and simple method, through dental explorer, without success. Therefore, the fragment was destroyed with a fissure bur, touching the internal threads of the implant, as seen radiographically (Figure 2).

**Figure 3:**
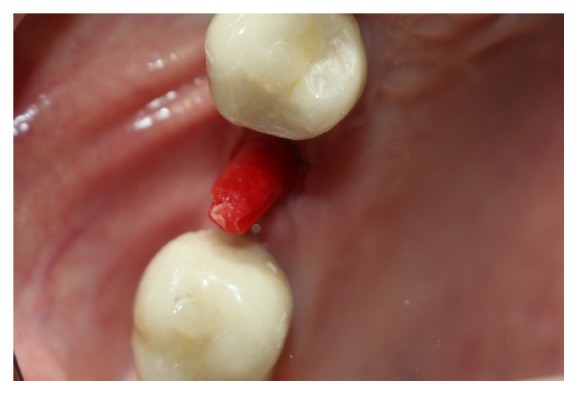
DuraLay post.

The gum around the implant platform was cut minimally and an acrylic resin impression made of the inside of the implant (DuraLay, Reliance Dental Mfg. Keliance) (Figure 3).

**Figure 4:**
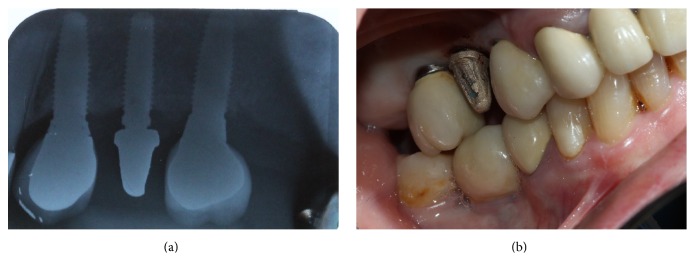
Sealing and adaptation were made: (a) radiographic control and (b) disclosing wax verifications.

**Figure 5:**
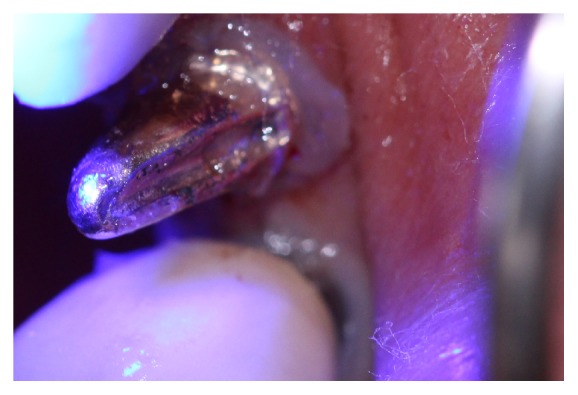
Post cement.

Once the post cast bolt (gold-palladium) was made and its adaptation with wax (Wax Disclosing Kerr) checked radiographically (Figure 4), it was cemented with the following protocol (Figure 5):

- The inside of the implant was cleaned, washed, and dried
- Hydrofluoric acid gel at 9.6% (EUFAR Laboratories S.A.) was applied to the post for 60 seconds, then washed, and dried
- Luting cement (DTK-Klever Bredent, DE) was mixed and placed both on the post and inside the implant
- The post was photopolymerized with ultraviolet light (350 to 500 Nm) for 3 minutes
- Excess cement was removed

**Figure 6:**
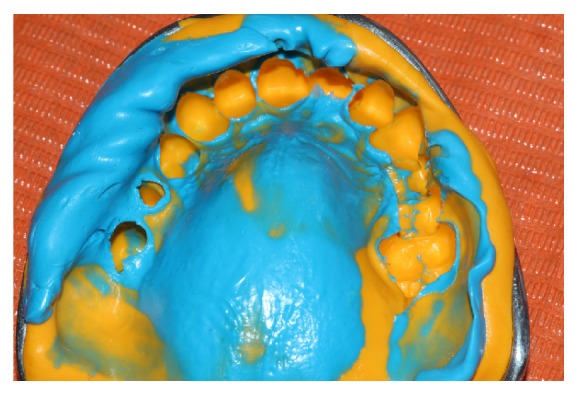
Dental impression.

**Figure 7:**
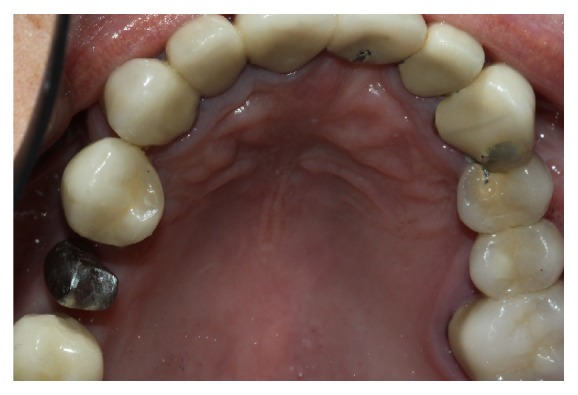
Framework sealing and adaptation of metal crown.

Once the post cemented, cord (Ultradent Ultrapak of two ESPA 00) is placed, printing was performed with heavy and light polyvinyl siloxane (Elite HD + from Zhermack IT) (Figure 6), and metal framework developed (silver-palladium) (Figure 7), by wax (wax Disclosing Kerr) adaptation, and sealing was made.

**Figure 8:**
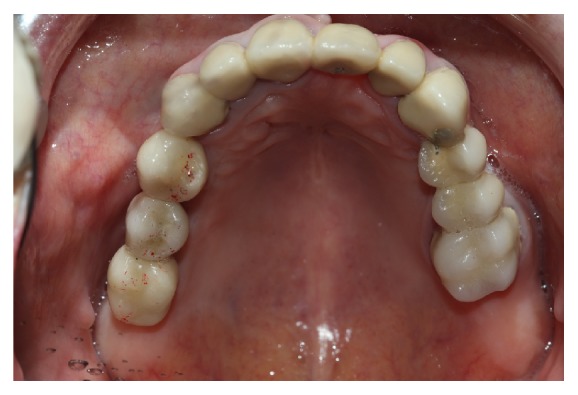
Crown cement and occlusal adjustment.

**Figure 9:**
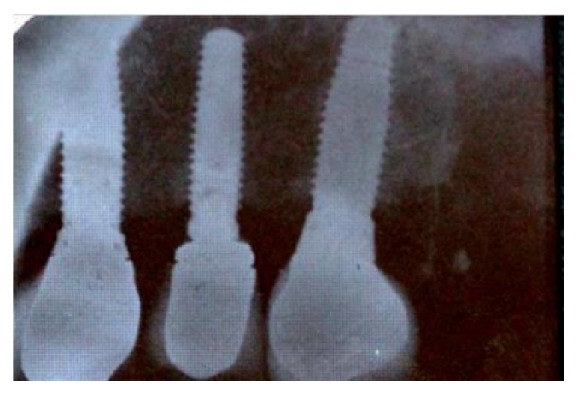
Intraoral radiograph at the 1-year follow-up.

Finally the prosthetic crown is cemented, after occlusal control, which was allowed to be below 12 micrometers' occlusion (Figure 8) and the patient received information about oral hygiene. After one year of treatment, clinical and radiographic monitoring is performed (Figure 9).

## 3. Discussion

To Hurson [12] screw loosening is a possible complication of the prosthesis screwed implants, leading to dissatisfaction for the patient and frustration for the dentist, and if left untreated it can lead to breakage thereof or one of the implant components becoming more complex and difficulty in solving mechanical complication, as in the present case. Rangert et al. [18] reported that 90% of fractured implants are in the region of the molars and premolars. Similar observations were made by Balshi [24], who found that all implant fractures occurred in the area of the premolars and molars with no distinction between upper and lower jaw. Van Steenberghe et al. [20] state that the first failures occur in the posterior maxilla, with a success rate of 91.4% compared to the previous maxilla with 97%.

In the article by Zarb and Schmitt [2] in which 225 implants were lost, 109 were lost after the prosthetic treatment and generally performed posteriorly. For Jemt [15] they were lost in 1.9% in individual crown on implants. While for Andersson et al. [7] and Haas et al. [8] the most common complication reported with single crowns was a pillar and/or prosthetic screw loosening. A higher frequency of screw loosening according to Ekfeldt et al. [5] and Laney et al. [6] was produced in individual crowns in premolars and molars compared to the previous region. Within the limits of a retrospective study, conducted for W. Becker and B. E. Becker [16] in replacement of molars for implants, it was found that the main complication was loosening gold screws, presented in eight implants (38%) of 21 implants, and one implant was lost and the survival rate was 95.7%.

Jemt [15] stated screws fractured by fatigue occur in the first year of operation, provided that the design of the prosthesis is not appropriate. To Schwarz [10] preload is the only resistance to occlusal forces in implant external hexagon with individual crowns. If occlusal forces exceed this preload, screw loosening and thus its fracture can be produced [11].

In this case, the patient had fracture of the prosthetic screw with damaged external hexagon but had noticed loosening of the screw several times before its fracture; however, it had been decided to maintain the implant within the mouth for further prosthetic rehabilitation as the patient was taking bisphosphonates. The treatment reported here is in contrast to Gargallo Albiol et al. [19] who conducted an analysis of fracture implants in which 81% was complete removal and subsequent placement of a greater number of implants and larger diameter in the region of premolars and molar. Satwalekar et al. [22] presented a case corresponding screw and fractured the left central incisor, which left it submerged after removing the fractured part implant; then a fixed partial denture was placed. Goiato et al. [25] reported a case of a 58-year-old with implant fractured in a cervical third level in the maxillary first premolar, which was removed trepano in the same clinical session. Three months later a new implant and prosthesis were put on.

It has been proposed to wear down or attempt to make a notch in the head fragment of the broken screw with a round bur at high speed, depending on the location of the prosthetic screw. In the case reported it was not possible to extract the fragment because it was below the implant head so it was ground down with a bur. The risk of puncturing the implant and bone was controlled radiographically. After achieving the objective, a metal and porcelain post and crown were made.

Among the advantages of using noble alloys is that they have a lower elastic modulus allowing the occlusal forces to transmit more efficiently to the remaining teeth. Binding of noble metal and porcelain is better than base metal because the oxide layer is thinner. Disadvantage is the high economic cost [26].

Finally, in a monitoring appointment after one year of treatment the patient reported no discomfort. Normality was clinically and radiographically checked.

## 4. Conclusion

Within the limitations of this case it was possible to conclude that the situations with screws fractured abutment and devastated hexagon can be solved in a time and manner of saving money, with a very useful alternative without the invasive treatment such as removal of the implant and submerging. Therefore the present technique used in this case is very simple, noninvasive, and useful to readers.
